# Longitudinal Regional Trends in Healthcare Capacity and Recorded Service Utilization in the Saudi Ministry of Health, 2016–2024

**DOI:** 10.3390/healthcare14183033

**Published:** 2026-09-16

**Authors:** Abdullah Bin Salamah, Nurah Alamro

**Affiliations:** 1 Department of Family and Community Medicine, College of Medicine, King Saud University, Riyadh 11461, Saudi Arabia; nmalamro@ksu.edu.sa; 2 Department of Family and Community Medicine, King Saud University Medical City, King Saud University, Riyadh 11461, Saudi Arabia

**Keywords:** Saudi Arabia, Ministry of Health, healthcare supply, recorded service utilization, population-adjusted rates, regional monitoring

## Abstract

**Background/Objectives:** Regional monitoring is needed to determine how Ministry of Health (MoH) healthcare supply and recorded service utilization changed during Saudi Arabia’s health-sector transformation. This descriptive study examines selected indicators relevant to Sustainable Development Goal 3 (SDG 3: good health and well-being). **Methods:** We conducted a retrospective longitudinal ecological study with descriptive analysis using data from all 13 administrative regions from 2016 to 2024. Eight MoH indicators were harmonized from statistical yearbooks and examined as absolute counts and population-adjusted rates. Period-specific comparisons, capacity-to-utilization ratios, and an analysis excluding the 2024 endpoint were also conducted. **Results:** Nationally, hospitals increased by 5.8%, beds by 12.1%, physicians by 78.1%, and nurses by 20.5%, whereas primary healthcare (PHC) centers declined by 6.6%. Recorded PHC encounters decreased by 17.2%, while outpatient department visits and inpatient admissions increased by 15.6% and 60.4%, respectively. After population adjustment, physicians per 10,000 population increased by 56.1%, nurses per 10,000 by 5.6%, and admissions per 1000 by 40.7%; beds per 1000 decreased by 1.7%, PHC encounters per person decreased by 27.4%, and outpatient visits per person increased by 1.4%. A marked change in recorded values occurred during 2020, and several measures changed sharply again in 2024. **Conclusions:** The selected MoH supply and recorded service utilization indicators followed different national and regional trajectories. The observed differences identify monitoring priorities; they do not establish causal effects or changes in access, equity, efficiency, or quality.

## 1. Introduction

Saudi Arabia’s health sector is undergoing simultaneous changes in organization, workforce, infrastructure, digital delivery, and care pathways under Vision 2030 and the Health Sector Transformation Program (HSTP) [1]. For planners, the immediate monitoring problem is whether Ministry of Health (MoH) healthcare supply and recorded service utilization are changing in comparable or divergent ways across administrative regions. National expansion in one indicator may coexist with limited change or decline in another, while national totals may conceal different regional trajectories.

The scale of demographic and service change makes this question consequential. The Kingdom’s mid-year population estimate increased from 30.95 million in 2016 to 35.30 million in 2024 (+14.0%) [2]. Over the same years, the harmonized MoH series examined here recorded increases of 78.1% in physicians, 20.5% in nurses, 12.1% in beds, and 5.8% in hospitals, together with a 6.6% decline in PHC centers [3]. Recorded service utilization also changed substantially: PHC encounters declined by 17.2%, whereas hospital outpatient visits and inpatient admissions increased by 15.6% and 60.4%, respectively [3]. These statistics describe selected MoH healthcare supply and recorded service utilization rather than the whole Saudi health system, but their differing magnitudes create a practical need for integrated regional monitoring.

The Saudi health-policy literature establishes several parts of this context. Primary healthcare is widely recognized as a foundation for accessible and integrated health services [4]. Al Asmri et al. described a public system and primary-care sector in transition, emphasizing persistent organizational and service-delivery challenges [5]. Alasiri and Mohammed reviewed post–Vision 2030 transformation and identified changes in governance, delivery models, privatization, and digital health [6]. Al-Nozha’s qualitative study showed that reform affects stakeholders through organizational, financing, and implementation changes that require careful management [7]. The World Bank labor-market assessment documented projected imbalances between physician and nursing supply, need, and demand [8], while the Saudi Health Council and Alnowibet et al. separately highlighted nursing challenges and changing healthcare human-resource requirements [9,10]. Collectively, this work demonstrates major PHC, workforce, and organizational change, but these domains have generally been examined separately or at one point in time.

The remaining gap is longitudinal and regional. Limited evidence has tracked selected MoH infrastructure, workforce, and recorded service utilization together across all 13 administrative regions throughout 2016–2024, while also addressing population growth, the marked 2020 change and the 2024 reporting transition. A harmonized regional series would provide a descriptive baseline for identifying trajectories that warrant closer operational investigation.

### 1.1. Problem Statement

Major expansion and restructuring of MoH healthcare capacity are occurring without a single longitudinal regional account showing how resource changes correspond descriptively with recorded service utilization in PHC and hospital settings. Because the population increased by 14.0% during the study period [2], absolute growth alone cannot show whether capacity or utilization kept pace with the population served.

This evidence gap affects MoH planners, health-cluster leaders, regional administrators, PHC leadership, hospital executives, workforce planners, and policymakers preparing post-2030 monitoring. Without comparable regional time series, national totals may conceal unusually rapid changes in recorded service volume, population-adjusted declines, or measurement discontinuities. Previous studies have addressed PHC, workforce, or reform separately [5,6,7,8,9,10]; the unresolved research need is to describe these selected supply and recorded service utilization trajectories together over time.

### 1.2. Originality, Rationale, and Significance of the Inquiry

This study addresses an underexamined area by harmonizing eight MoH indicators for every administrative region across nine consecutive years. Its contribution is the combination of national and regional absolute trends, annual population-adjusted rates, period-specific comparisons, capacity-to-utilization ratios, and explicit sensitivity to the 2024 reporting transition.

The rationale is that supply and recorded service utilization describe different aspects of service delivery. Resource counts do not establish how services were used, and utilization counts do not establish whether capacity was adequate or care appropriate. Examining both domains in parallel can identify divergence and abrupt changes requiring investigation, while preserving the distinction between description and explanation.

The study provides a longitudinal regional baseline connecting Saudi PHC, workforce, and reform research. Managers can use it to locate indicators and regions requiring source review or service-level assessment. It also supports the development of monitoring systems that combine absolute volume and population denominators with setting-specific measures, digital and non-MoH activity, quality, and outcomes.

### 1.3. Literature Review and Conceptual Framework

Three complementary frameworks define the evidentiary scope of this analysis. Donabedian distinguishes healthcare structure, process, and outcomes [11]. In the present study, PHC centers, hospitals, beds, physicians, and nurses are structural indicators, while recorded PHC encounters, outpatient visits, and inpatient admissions are measures of activity related to care processes. Clinical outcomes and quality are outside the measured domains.

The Andersen Behavioral Model emphasizes that health-service use reflects not only enabling resources but also population characteristics, need, socioeconomic conditions, preferences, financing, and accessibility [12]. This provides a rationale for population adjustment and for considering influences beyond the measured supply indicators. The WHO health-system building-blocks framework places workforce and service delivery within a wider system that also includes information, medicines and technologies, financing, and leadership/governance [13]. The present indicators cover only part of that framework.

Applied to Saudi Arabia, these frameworks integrate the empirical literature. Studies of PHC transition [5], system transformation [6], stakeholder experience [7], workforce supply and demand [8,9,10], and integrated SDG 3 measurement [14] collectively show that reform affects interacting components. They do not, however, provide a harmonized annual regional account of how the selected healthcare supply and recorded service utilization indicators changed together. The present study addresses this gap by comparing the selected indicators across regions and years.

### 1.4. Study Objective

The primary objective was to describe and compare annual changes in selected MoH healthcare supply and recorded service utilization indicators across Saudi Arabia’s 13 administrative regions from 2016 to 2024. Secondary descriptive objectives were to compare absolute and population-adjusted trajectories, calculate setting-relevant capacity-to-utilization ratios, distinguish pre-disruption, 2020, and subsequent periods, and assess whether the principal findings were sensitive to exclusion of the 2024 endpoint.

## 2. Materials and Methods

### 2.1. Study Design and Setting

This was a retrospective longitudinal ecological study with descriptive analysis using a balanced region–year dataset. Aggregated data covered all 13 administrative regions annually from 2016 through 2024 (117 region–years). The dataset therefore comprised repeated observations of the same administrative regions over time; no inferential modeling or causal analysis was undertaken. The empirical scope was restricted to MoH facilities, workforce, and recorded services and does not represent private, university, military, or other governmental providers.

### 2.2. Data Sources and Extraction

Eight indicators were obtained from Saudi MoH Statistical Yearbooks for 2016–2024 [3]. Excel workbooks were used for 2016, 2019, 2020, 2023, and 2024; PDF yearbooks were used for 2017, 2018, 2021, and 2022, as documented in the extraction log. Excel tables were imported programmatically where the tabular structure permitted. PDF tables were manually transcribed into standardized indicator worksheets and visually checked against the cited source tables. The extraction log records the year, indicator, table, chapter, source file, and relevant construction note for every series.

Verification included comparison of each structured row with its cited source table, arithmetic checks of regional sums against reported national totals where available, range and adjacent-year review, and reinspection of the original table whenever a discrepancy was identified. Workforce corrections and their source-based resolution are recorded in a separate correction log. No independent duplicate extraction was performed. The harmonized data, extraction and correction logs, indicator rules, and analysis code are supplied to allow readers to check reported values and derivations.

### 2.3. Indicators and Construction Rules

Supply indicators were the number of MoH PHC centers, MoH hospitals, MoH hospital beds, physicians, and nurses. Recorded service utilization indicators were PHC encounters, outpatient department (OPD) visits at MoH hospitals, and inpatient admissions at MoH hospitals. Physician counts were defined as hospital physicians minus hospital dentists plus PHC physicians minus PHC dentists. Nurse counts were hospital nurses plus PHC nurses. These workforce measures combine hospital and PHC personnel and therefore cannot be attributed to either setting separately. The source yearbooks report administrative headcounts/categories rather than a consistently identified full-time-equivalent measure and do not permit consistent identification of residents, locums, contracts, or possible cross-setting duplication.

The high-level service labels were traceable across the nine yearbooks, but detailed reporting did not allow consistent separation of physical and virtual PHC encounters. Consequently, temporal changes in PHC or OPD series may reflect changes in delivery mode, organizational classification, or reporting practice as well as changes in recorded service utilization. A supplementary indicator dictionary records the construction rule, setting, denominator, and comparability limitation for each variable.

### 2.4. Regional Harmonization and 2024 Reporting Geography

All values were harmonized to the 13 administrative regions. The official 2024 reporting-unit crosswalk was taken from the MoH Statistical Yearbook 2024 workbook, worksheet ‘Regions—English’ (English translation of the worksheet name), cells B1:F25, titled ‘Former Health Affairs Directorates and their Corresponding Administrative Regions, Governorates, Health Clusters, and Branches & Offices of the Ministry of Health’ [3]. Appendix A provides the indicator-specific source worksheets and the complete crosswalk.

PHC centers used MoH Statical Yearbook 2024 worksheet 2-5; hospitals and beds used worksheet 2-6; hospital and PHC workforce used worksheets 2-41 and 2-44; PHC encounters and OPD visits used worksheets 4-6 and 4-7; and inpatient admissions used worksheet 4-22. Every named 2024 branch, office, or health cluster used in the final regional dataset was assigned to one administrative region only. The 16,783 PHC encounters labeled ‘Undefined’ in the 2024 source could not be assigned to a region and were excluded from the regional analytic total; the corresponding undefined OPD value was zero. Supplementary File S1 flags and documents the 2024 transformation. No statistical imputation was used.

### 2.5. Population Denominators

Annual regional and Kingdom mid-year population estimates for 2016–2024 were obtained from the KAPSARC Data Portal dataset ‘Population by Administrative Region and Gender,’ created by the Saudi Central Bank (SAMA); total population (Gender = Total) was used [2]. The sum of the 13 regional values equaled the published Kingdom total in every year. Rates were calculated as PHC centers and hospitals per 100,000 population, beds and inpatient admissions per 1000 population, physicians and nurses per 10,000 population, and PHC encounters and OPD visits per person. Each population-adjusted rate was calculated by dividing the regional or national indicator count by the corresponding mid-year population and multiplying by the applicable scale factor of 100,000, 10,000, or 1000. PHC encounters and OPD visits per person were calculated by dividing the corresponding count directly by the population. These rates standardize for population size but not age, morbidity, socioeconomic conditions, geographic accessibility, or healthcare need.

### 2.6. Statistical Analysis

The analysis was exclusively descriptive. Absolute change was calculated as the endpoint value minus the baseline value. Percentage change was calculated as [(endpoint value − baseline value)/baseline value] × 100. Count change (%) and rate change (%) were calculated using the same percentage-change formula applied to the corresponding absolute counts and population-adjusted rates, respectively. CAGR was calculated as [(2024 value/2016 value)^1/8^ − 1] × 100, and indexed values were calculated as (annual value/2016 value) × 100. For every indicator, we calculated annual national totals, regional values, absolute and percentage changes from 2016 to 2024, compound annual growth rates (CAGRs), and indexed trends (2016 = 100). Absolute counts represent MoH system volume; population-adjusted rates represent supply or recorded service utilization relative to the resident population denominator. Regional heatmaps used a diverging scale centered at zero, and exact values remained visible in the tables and cell labels.

Each capacity-to-utilization ratio was calculated by dividing the recorded service-utilization count by the corresponding facility or bed count for the same year: PHC encounters by PHC centers, OPD visits by MoH hospitals, and inpatient admissions by MoH beds. Average length of stay and bed occupancy were not added because comparable regional annual series were not available across all study years.

Period-specific percentage changes were calculated for 2016–2019 (pre-disruption), 2019–2020 (marked 2020 change), 2020–2024 (recovery/reconfiguration), and 2019–2024 (comparison with the immediate pre-2020 level). Period-specific and endpoint percentage changes were calculated using the same percentage-change formula with the relevant start and endpoint years. The labels describe timing only and do not attribute the marked 2020 change exclusively to the pandemic. A descriptive endpoint sensitivity analysis repeated the principal comparison for 2016–2023 and contrasted it with 2016–2024, with particular attention to physicians and inpatient admissions because of their large 2024 changes.

All analyses were conducted using Python 3.12.13. The harmonized analytic data, extraction and correction logs, construction rules, and executable code are supplied with the manuscript. No inferential modeling was performed.

### 2.7. Ethical Considerations

Only publicly available, aggregated administrative data were used; no individual-level or identifiable human participant information was analyzed. The Institutional determination is reported in the Institutional Review Board Statement. This study is reported in accordance with the STROBE guidance for observational studies, where applicable.

## 3. Results

### 3.1. Data Completeness and Comparability

All 117 region–years had recorded values after extraction, arithmetic derivation, and geographic harmonization. Some utilization entries published in the 2017 and 2019 yearbooks were explicitly labeled as previous-year data; these values were retained and are identified in Supplementary File S1. Derived workforce values and 2024 re-aggregations are also documented there. No additional imputation was applied by the authors. Regional population denominators were complete for all 117 region–years.

### 3.2. National Absolute and Population-Adjusted Trends

**Figure 1:**
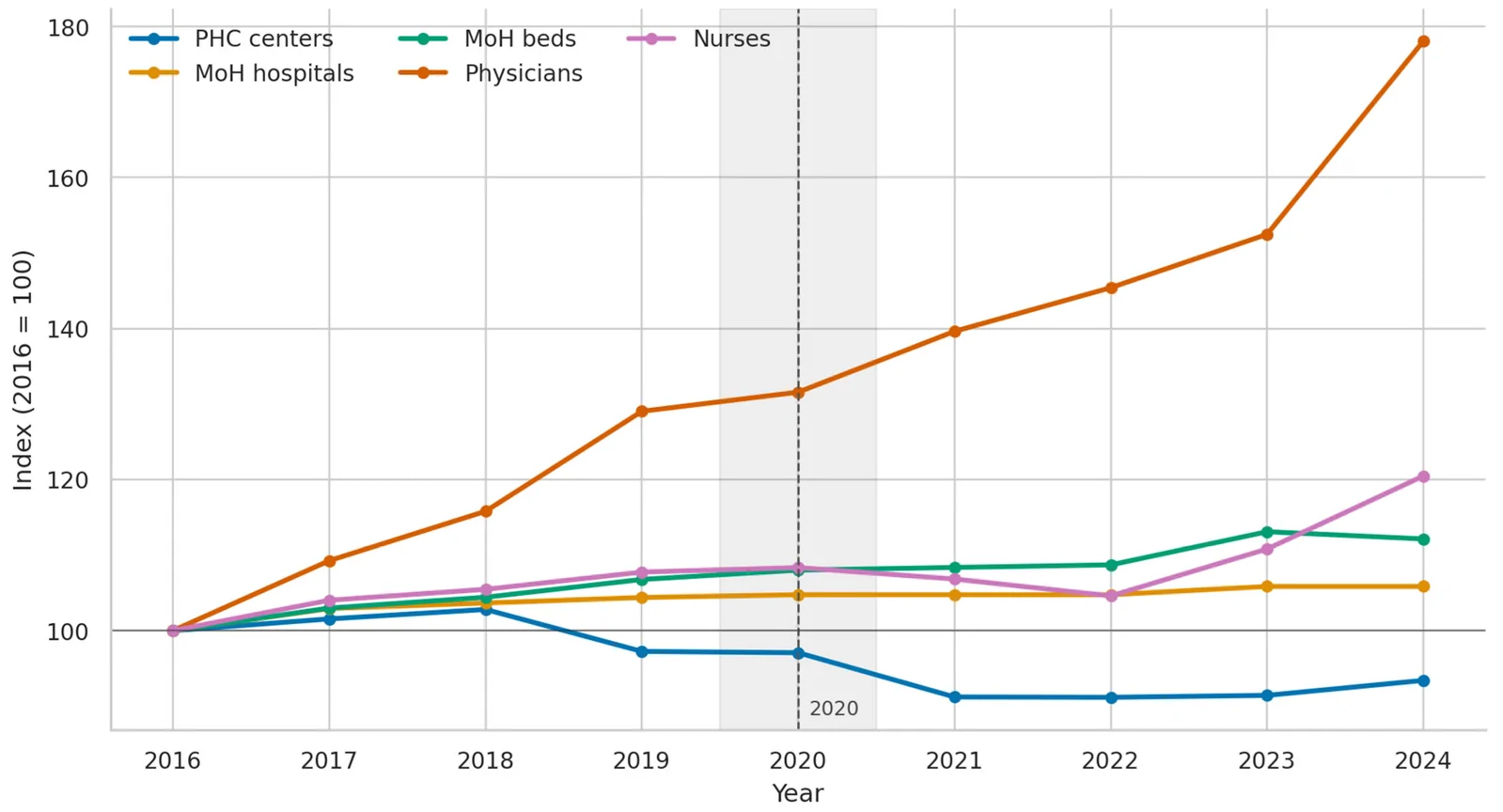
Indexed national MoH supply indicators, 2016–2024 (2016 = 100). The shaded band identifies the marked change in recorded values during 2020; it does not represent a causal estimate.

**Figure 2:**
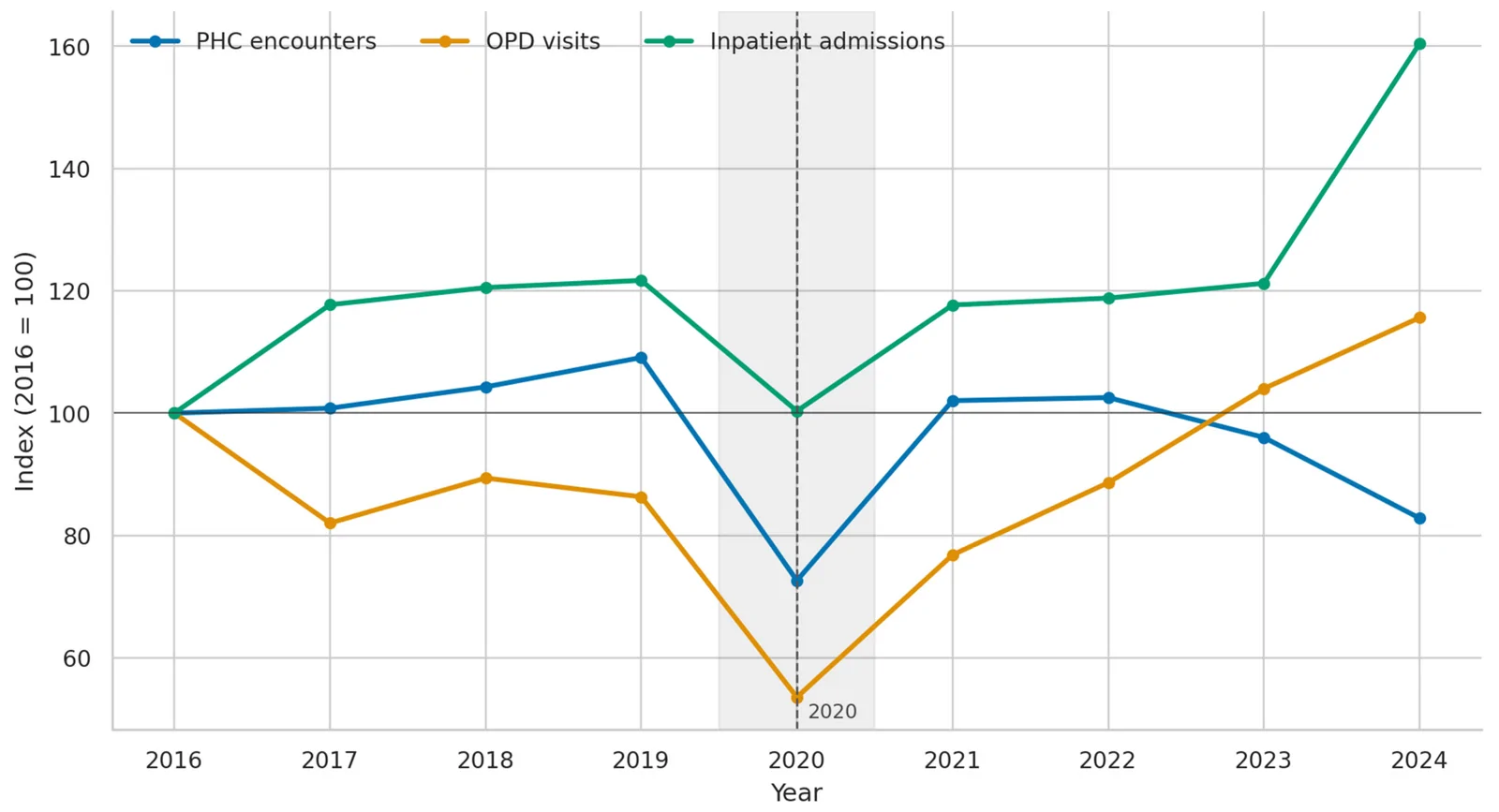
Indexed national MoH recorded service utilization indicators, 2016–2024 (2016 = 100). The shaded band identifies the marked change in recorded values during 2020; it does not represent a causal estimate.

From 2016 to 2024, MoH hospitals increased from 274 to 290 (+5.8%), beds from 41,835 to 46,905 (+12.1%), physicians from 37,854 to 67,403 (+78.1%), and nurses from 99,224 to 119,530 (+20.5%). PHC centers declined from 2325 to 2172 (−6.6%). Recorded PHC encounters decreased from 49,817,811 to 41,258,833 (−17.2%), whereas OPD visits increased from 14,529,099 to 16,796,225 (+15.6%) and inpatient admissions from 1,169,972 to 1,876,723 (+60.4%) (Table 1; Figure 1 and Figure 2).

The population increased from 30,954,198 to 35,300,280 (+14.0%). After adjustment, PHC centers per 100,000 population decreased by 18.1%, hospitals per 100,000 by 7.2%, and beds per 1000 by 1.7%. Physicians per 10,000 increased by 56.1% and nurses per 10,000 by 5.6%. PHC encounters per person decreased by 27.4%, OPD visits per person increased by 1.4%, and inpatient admissions per 1000 increased by 40.7% (Table 1). Thus, several absolute increases were attenuated or reversed after accounting for population growth.

### 3.3. Regional Variation

**Figure 3:**
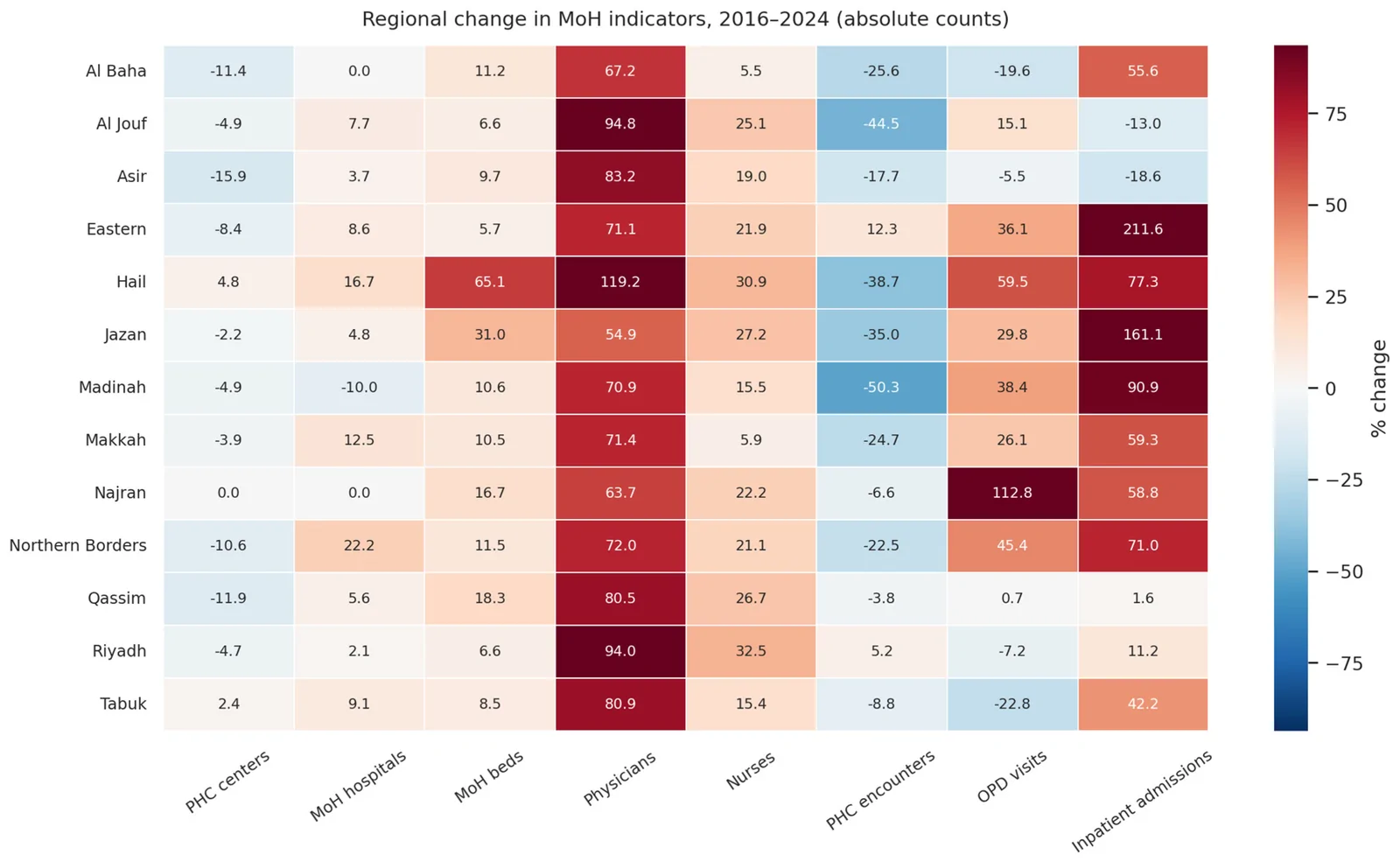
Regional percentage change in all eight MoH indicators, 2016–2024, based on absolute counts. The diverging scale is centered at zero; exact values label each cell.

Regional changes were heterogeneous across all eight indicators (Table 2 and Table 3; Figure 3). Physician growth ranged from +54.9% in Jazan to +119.2% in Hail, and nurse growth ranged from +5.5% in Al Baha to +32.5% in Riyadh. PHC encounters declined most in Madinah (5,419,539 to 2,693,038; −50.3%) but increased in Eastern (7,388,187 to 8,296,654; +12.3%). OPD visits increased most in relative terms in Najran (292,824 to 623,018; +112.8%). Inpatient admissions increased from 139,360 to 434,299 in Eastern (+211.6%) and from 59,140 to 154,405 in Jazan (+161.1%); presenting the starting and ending counts makes the scale of these percentages explicit.

**Figure 4:**
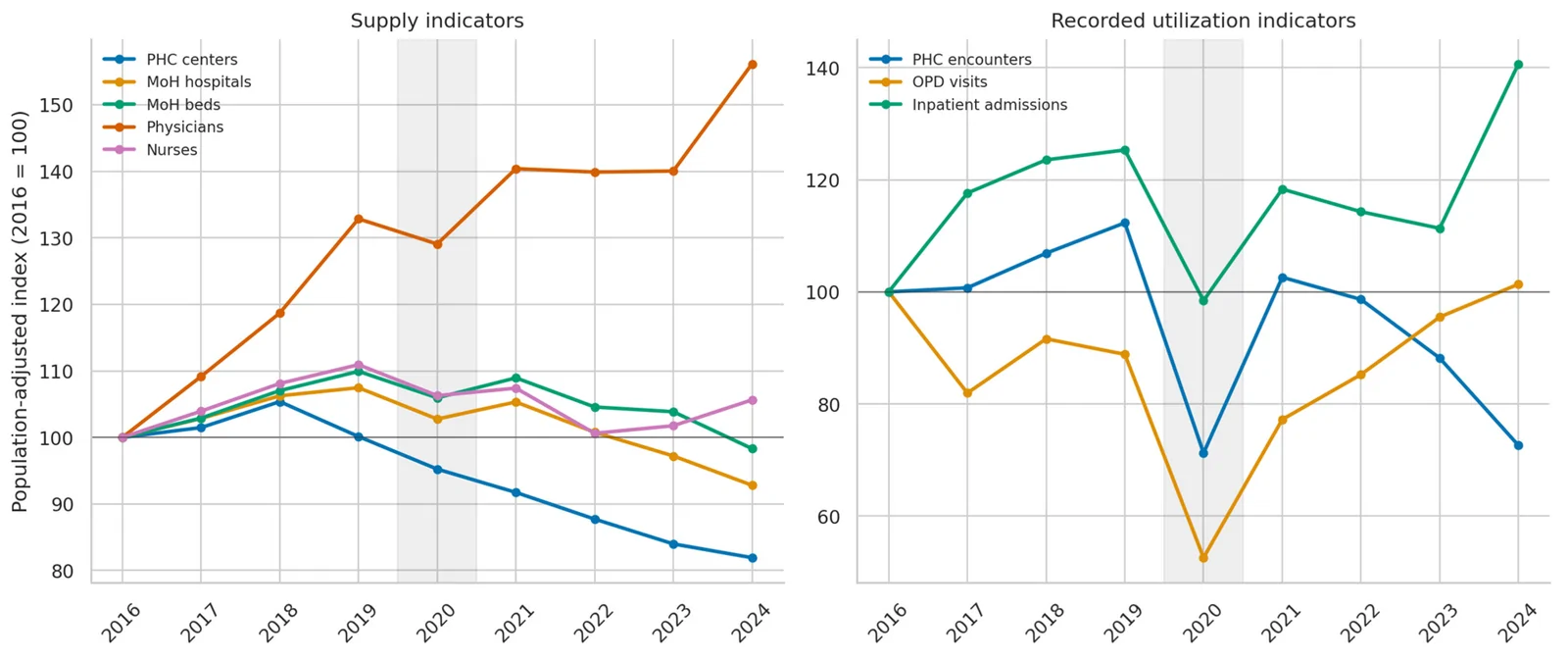
Indexed national population-adjusted rates for MoH indicators, 2016–2024 (2016 = 100). Supply and recorded service utilization indicators are displayed separately. The shaded band identifies the marked change in recorded values during 2020; it does not represent a causal estimate.

**Figure 5:**
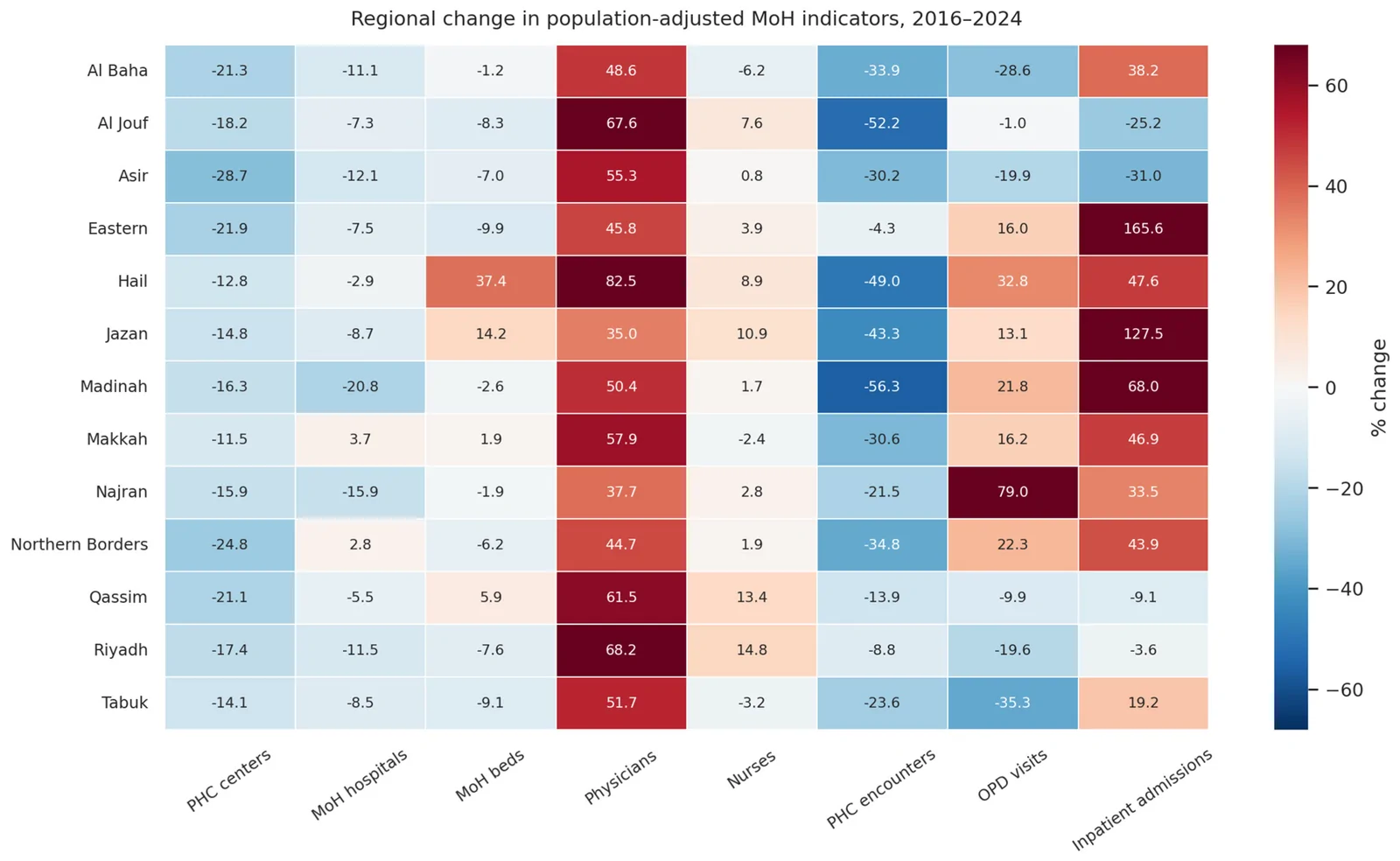
Regional percentage change in population-adjusted rates for MoH indicators, 2016–2024. The diverging scale is centered at zero; exact values label each cell. Rates do not adjust for age, morbidity, density, or need.

Population adjustment changed the interpretation of some national and regional trajectories (Figure 4 and Figure 5). For example, Eastern PHC encounters increased by 12.3% in absolute count but decreased by 4.3% per person. Physicians per 10,000 population increased in every region, ranging from +35.0% in Jazan to +82.5% in Hail. In contrast, population-adjusted PHC encounters declined in every region, ranging from −4.3% in Eastern to −56.3% in Madinah.

### 3.4. Period-Specific Changes in 2020 and Surrounding Periods

The 2016–2024 interval was not homogeneous (Table 4). Before 2020, from 2016 to 2019, PHC encounters increased by 9.1%, OPD visits decreased by 13.7%, and inpatient admissions increased by 21.7%. From 2019 to 2020, the corresponding recorded volumes declined by 33.4%, 37.9%, and 17.6%. These simultaneous changes show a marked change in recorded activity during 2020 but cannot distinguish pandemic effects from reporting changes, digital delivery, service reorganization, or substitution outside the observed MoH sector.

From 2020 to 2024, PHC encounters increased by 14.0%, OPD visits by 115.7%, and inpatient admissions by 59.9%. Relative to 2019, however, 2024 PHC encounters remained 24.1% lower, while OPD visits were 33.9% higher and inpatient admissions 31.8% higher. The population-adjusted 2019–2024 changes were −35.3%, +14.1%, and +12.3%, respectively. Accordingly, ‘recovery’ refers only to change from the 2020 recorded level and not to restoration of the same service-delivery or reporting construct.

### 3.5. Capacity-to-Utilization Ratios

**Figure 6:**
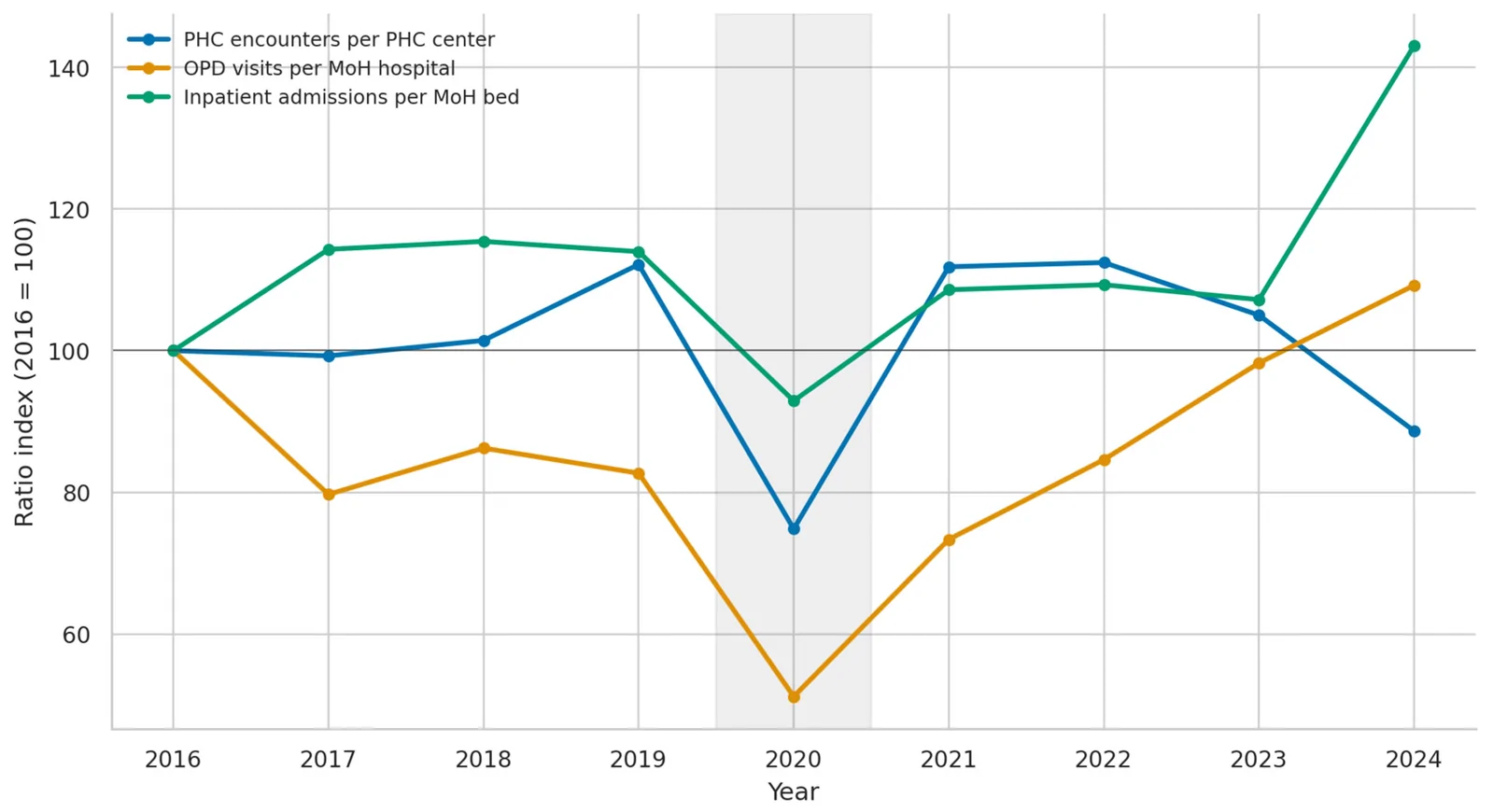
Indexed national capacity-to-utilization ratios, 2016–2024 (2016 = 100). The shaded band identifies the marked change in recorded values during 2020; it does not represent a causal estimate.

PHC encounters per PHC center declined from 21,427 in 2016 to 18,996 in 2024 (−11.3%). OPD visits per MoH hospital increased from 53,026 to 57,918 (+9.2%). Inpatient admissions per MoH bed increased from 28.0 to 40.0 (+43.1%), with the sharpest endpoint change occurring in 2024 (Table 5; Figure 6). Without length of stay, occupancy, case mix, or admission-threshold data, the admissions-per-bed increase cannot be classified as improved efficiency or excessive pressure.

### 3.6. Sensitivity to Exclusion of 2024

Excluding 2024 preserved the direction of change for all eight indicators but materially reduced several magnitudes (Table 6). Through 2023, physicians had increased by 52.5% rather than 78.1%, and inpatient admissions by 21.2% rather than 60.4%. The 2023–2024 changes were +16.8% for physicians and +32.3% for admissions. PHC encounters were 4.0% below 2016 in 2023 and 17.2% below in 2024. Different trajectories across indicators were already evident through 2023, but estimates that depend strongly on the 2024 endpoint require particular caution.

## 4. Discussion

### 4.1. Principal Findings

This longitudinal ecological study provides descriptive monitoring of selected MoH healthcare supply and recorded service utilization indicators. Physician counts and physicians per 10,000 population increased substantially, whereas changes in hospitals, beds, and nurses were smaller after population adjustment. PHC centers and recorded PHC encounters declined in both absolute and population-adjusted terms. Recorded OPD visits changed little per person, while inpatient admissions increased on both measures. These national patterns varied by region and were interrupted in 2020; large changes in 2024 also affected the endpoint comparisons.

The selected supply and recorded service utilization indicators followed different trajectories across settings, regions, and periods. The descriptive comparisons cannot establish independent effects of individual supply indicators on utilization.

### 4.2. Interpretation in Relation to the Conceptual Framework and the Previous Literature

In Donabedian’s framework, the study documents substantial change in selected structural inputs and in activity measures related to process, while leaving outcomes unmeasured [11]. This distinction is important because the 78.1% growth in physicians cannot be treated as evidence of improved care on its own. The workforce trajectory rose across most years, reaching +52.5% by 2023, followed by a further 16.8% increase in 2024. The supplied extraction and correction logs document the validated indicator data and year-by-year source verification; the available aggregate data cannot distinguish genuine workforce expansion from changes in classification, reporting coverage, organizational attribution, or a combination of these mechanisms. The endpoint sensitivity analysis therefore supports the direction of workforce growth while qualifying its exact magnitude.

The Andersen model also cautions against interpreting supply and utilization as a simple input–output relationship [12]. Population, need, preferences, insurance, geographic accessibility, and alternative providers can affect recorded service use. Population adjustment strengthened some contrasts: hospital counts grew by 5.8% but hospitals per 100,000 population declined by 7.2%; beds grew by 12.1% but beds per 1000 declined by 1.7%; and OPD visits grew by 15.6% but visits per person increased by only 1.4%. These differences show why regional monitoring needs both absolute volumes and population denominators.

The findings extend the prior Saudi literature by bringing previously separate domains into one regional time series. Earlier work described PHC challenges [5], organizational transformation [6,7], and workforce supply and demand [8,9,10]. The present analysis shows that substantial workforce growth, a decline in recorded PHC service utilization, an increase in recorded hospital service utilization, and geographic heterogeneity coexisted over the same period.

### 4.3. PHC, Hospital Utilization, and Measurement

The decline in recorded PHC encounters is the most persistent utilization signal. Encounters fell by 17.2% from 2016 and by 24.1% from the 2019 pre-2020 level; per-person encounters fell by 27.4% and 35.3%, respectively. Interpretation requires attention to physical and virtual service delivery, referral redesign, organizational reclassification, reporting changes, patient preferences, and movement to private or other non-MoH providers. Because the yearbooks do not consistently separate virtual encounters, this is a measurement limitation as well as a possible substantive explanation.

Inpatient admissions increased more rapidly than beds. Admissions per bed rose by 43.1%, and Eastern and Jazan recorded particularly large endpoint increases. These ratios warrant operational examination, but several distinct mechanisms could explain this pattern, including shorter stays, higher turnover, different case mix, changed admission thresholds, changes in service demand, or reporting changes. Comparable annual regional occupancy and length-of-stay data were unavailable, so no efficiency or capacity-adequacy conclusion is drawn.

The 2020 declines are presented as a marked change in recorded values rather than a uniquely identified COVID-19 effect. The design cannot separate pandemic restrictions and care avoidance from reporting changes, digital delivery, service reorganization, or sectoral substitution. The different 2020–2024 trajectories therefore describe recovery or reconfiguration in recorded values, not necessarily recovery of the same underlying service construct.

### 4.4. Implications for Monitoring

Transparent descriptive monitoring should combine absolute counts, population-adjusted rates, and annual regional series. Absolute counts describe operational volume, while population-adjusted rates show whether that volume changed relative to population size. Annual regional series can identify abrupt changes hidden by national endpoints, although none of these measures explains why the changes occurred.

The relationship to SDG 3 is contextual [15]. The indicators represent selected workforce and service-delivery capacity and recorded service utilization relevant to the operational foundations of good health and well-being [13,16,17,18,19]. They do not directly measure universal health coverage, effective coverage, financial protection, mortality, quality, or equity. Post-2030 monitoring would be strengthened by linking these descriptive series to those outcomes and to private, digital, and other non-MoH services.

### 4.5. Limitations

The regional ecological design limits inference to aggregate patterns. Population-adjusted rates account for population size but leave age, morbidity, socioeconomic conditions, density, travel time, and other determinants of need unmeasured. Individual healthcare need, patient behavior, and realized access cannot be inferred from these regional measures.

Coverage is restricted to the MoH sector. Changes in private, university, military, or other governmental activity may alter recorded service utilization within the MoH sector without a corresponding change in total healthcare use. The yearbooks also span mixed file formats and organizational changes. Some utilization figures explicitly refer to previous-year data, preventing measurement of the actual annual change in affected observations. The 2024 source uses branches, offices, clusters, and administrative-region tables, and re-aggregation cannot eliminate all classification uncertainty.

The combined physician and nurse measures cannot be separated reliably into PHC and hospital workforce across all nine years, and the source does not consistently identify full-time equivalents or all employment categories. PHC and OPD definitions also prevent consistent separation of virtual and in-person activity. The undefined PHC category in 2024 could not be assigned to a region and was excluded from regional analysis.

Measures of quality, effective coverage, waiting time, mortality, readmissions, patient safety, average length of stay, bed occupancy, expenditure, case mix, and health outcomes were unavailable in the analytic dataset. Capacity-to-utilization ratios therefore describe recorded service utilization relative to capacity, without establishing efficiency or performance. The design cannot determine the causes of the observed trends or their implications for access, equity, quality, or population health.

## 5. Conclusions

This descriptive analysis of Saudi Arabia’s MoH sector from 2016 to 2024 found different trajectories in the selected healthcare supply and recorded service utilization indicators. Physician growth was substantial; hospital and bed growth was more modest and did not keep pace with population growth; PHC centers and recorded PHC encounters declined; recorded hospital service utilization increased; and regional trajectories differed. A pronounced change in recorded values during 2020 and large 2024 changes, particularly for physicians and admissions, require the endpoint estimates to be interpreted cautiously.

These results extend prior Saudi research by showing that PHC, workforce, infrastructure, and hospital activity trends coexisted rather than evolving as a single reform indicator. The practical implication is that isolated resource counts and national averages are insufficient for identifying which measures require source verification, setting-specific interpretation, or local operational investigation.

For future MoH monitoring and post-2030 planning, the following actions are recommended:

1. Develop integrated regional dashboards that display healthcare supply and recorded service utilization together.
2. Report annual regional population-adjusted rates alongside absolute operational volumes.
3. Establish focused PHC monitoring that distinguishes physical visits, virtual encounters, referral redesign, reporting changes, and movement to other sectors.
4. Retain PHC and hospital workforce separately whenever consistent source definitions permit.
5. Audit regions and years with abrupt changes before assigning substantive explanations or planning responses.
6. Integrate private-sector and other non-MoH activity to distinguish sectoral substitution from changes in overall use.
7. Add quality, outcome, need, and efficiency measures—including occupancy, length of stay, readmissions, waiting times, safety, and effective coverage—before assessing performance.
8. Maintain a versioned, official crosswalk and indicator dictionary whenever reporting boundaries or definitions change.

These priorities would allow future monitoring to examine how the observed trends relate to service coverage and patient outcomes.

## Figures and Tables

**Table 1:** National absolute and population-adjusted change in MoH indicators, 2016–2024.

Indicator	2016 Count	2024 Count	Absolute Change	Count Change (%)	2016 Rate	2024 Rate	Rate Change (%)
PHC centers	2325	2172	−153	−6.6%	7.511	6.153	−18.1%
MoH hospitals	274	290	+16	+5.8%	0.885	0.822	−7.2%
MoH beds	41,835	46,905	+5070	+12.1%	1.352	1.329	−1.7%
Physicians	37,854	67,403	+29,549	+78.1%	12.229	19.094	+56.1%
Nurses	99,224	119,530	+20,306	+20.5%	32.055	33.861	+5.6%
PHC encounters	49,817,811	41,258,833	−8,558,978	−17.2%	1.609	1.169	−27.4%
OPD visits	14,529,099	16,796,225	+2,267,126	+15.6%	0.469	0.476	+1.4%
Inpatient admissions	1,169,972	1,876,723	+706,751	+60.4%	37.797	53.165	+40.7%

Note: Rate units are PHC centers and hospitals per 100,000 population; beds and inpatient admissions per 1000; physicians and nurses per 10,000; and PHC encounters and OPD visits per person. Counts are sums of the 13 regional values in Supplementary File S1. Count CAGRs for 2016–2024 are reported in Supplementary File S1. MoH = Ministry of Health; PHC = primary healthcare; OPD = outpatient department. Rate changes were calculated from unrounded values.

**Table 2:** Regional absolute and percentage change in MoH supply indicators, 2016–2024.

Indicator	Region	2016 Count	2024 Count	Absolute Change	Count Change (%)
PHC centers	Al Baha	105	93	−12	−11.4%
PHC centers	Al Jouf	61	58	−3	−4.9%
PHC centers	Asir	334	281	−53	−15.9%
PHC centers	Eastern	250	229	−21	−8.4%
PHC centers	Hail	105	110	+5	+4.8%
PHC centers	Jazan	178	174	−4	−2.2%
PHC centers	Madinah	162	154	−8	−4.9%
PHC centers	Makkah	331	318	−13	−3.9%
PHC centers	Najran	68	68	0	0.0%
PHC centers	Northern Borders	47	42	−5	−10.6%
PHC centers	Qassim	177	156	−21	−11.9%
PHC centers	Riyadh	424	404	−20	−4.7%
PHC centers	Tabuk	83	85	+2	+2.4%
MoH hospitals	Al Baha	10	10	0	0.0%
MoH hospitals	Al Jouf	13	14	+1	+7.7%
MoH hospitals	Asir	27	28	+1	+3.7%
MoH hospitals	Eastern	35	38	+3	+8.6%
MoH hospitals	Hail	12	14	+2	+16.7%
MoH hospitals	Jazan	21	22	+1	+4.8%
MoH hospitals	Madinah	20	18	−2	−10.0%
MoH hospitals	Makkah	40	45	+5	+12.5%
MoH hospitals	Najran	11	11	0	0.0%
MoH hospitals	Northern Borders	9	11	+2	+22.2%
MoH hospitals	Qassim	18	19	+1	+5.6%
MoH hospitals	Riyadh	47	48	+1	+2.1%
MoH hospitals	Tabuk	11	12	+1	+9.1%
MoH beds	Al Baha	1165	1295	+130	+11.2%
MoH beds	Al Jouf	1820	1940	+120	+6.6%
MoH beds	Asir	3100	3400	+300	+9.7%
MoH beds	Eastern	6161	6511	+350	+5.7%
MoH beds	Hail	1175	1940	+765	+65.1%
MoH beds	Jazan	2225	2915	+690	+31.0%
MoH beds	Madinah	2818	3118	+300	+10.6%
MoH beds	Makkah	8345	9225	+880	+10.5%
MoH beds	Najran	1200	1400	+200	+16.7%
MoH beds	Northern Borders	1310	1460	+150	+11.5%
MoH beds	Qassim	2809	3324	+515	+18.3%
MoH beds	Riyadh	7937	8457	+520	+6.6%
MoH beds	Tabuk	1770	1920	+150	+8.5%
Physicians	Al Baha	1029	1721	+692	+67.2%
Physicians	Al Jouf	1110	2162	+1052	+94.8%
Physicians	Asir	2724	4991	+2267	+83.2%
Physicians	Eastern	5873	10,050	+4177	+71.1%
Physicians	Hail	967	2120	+1153	+119.2%
Physicians	Jazan	2114	3275	+1161	+54.9%
Physicians	Madinah	2805	4794	+1989	+70.9%
Physicians	Makkah	8431	14,449	+6018	+71.4%
Physicians	Najran	1037	1698	+661	+63.7%
Physicians	Northern Borders	918	1579	+661	+72.0%
Physicians	Qassim	2289	4131	+1842	+80.5%
Physicians	Riyadh	7283	14,128	+6845	+94.0%
Physicians	Tabuk	1274	2305	+1031	+80.9%
Nurses	Al Baha	2363	2493	+130	+5.5%
Nurses	Al Jouf	4238	5301	+1063	+25.1%
Nurses	Asir	6809	8100	+1291	+19.0%
Nurses	Eastern	15,029	18,327	+3298	+21.9%
Nurses	Hail	3072	4021	+949	+30.9%
Nurses	Jazan	5133	6531	+1398	+27.2%
Nurses	Madinah	7098	8199	+1101	+15.5%
Nurses	Makkah	20,126	21,308	+1182	+5.9%
Nurses	Najran	3091	3777	+686	+22.2%
Nurses	Northern Borders	2606	3156	+550	+21.1%
Nurses	Qassim	5880	7452	+1572	+26.7%
Nurses	Riyadh	20,032	26,540	+6508	+32.5%
Nurses	Tabuk	3747	4325	+578	+15.4%

Note: All values are counts. Detailed annual values and population-adjusted rates are supplied in Supplementary File S1.

**Table 3:** Regional absolute and percentage change in recorded service utilization indicators, 2016–2024.

Indicator	Region	2016 Count	2024 Count	Absolute Change	Count Change (%)
PHC encounters	Al Baha	1,494,684	1,112,303	−382,381	−25.6%
PHC encounters	Al Jouf	1,275,876	708,270	−567,606	−44.5%
PHC encounters	Asir	4,237,197	3,487,754	−749,443	−17.7%
PHC encounters	Eastern	7,388,187	8,296,654	+908,467	+12.3%
PHC encounters	Hail	1,762,725	1,080,922	−681,803	−38.7%
PHC encounters	Jazan	4,928,595	3,204,800	−1,723,795	−35.0%
PHC encounters	Madinah	5,419,539	2,693,038	−2,726,501	−50.3%
PHC encounters	Makkah	9,745,033	7,342,873	−2,402,160	−24.7%
PHC encounters	Najran	1,245,150	1,162,712	−82,438	−6.6%
PHC encounters	Northern Borders	1,013,350	785,480	−227,870	−22.5%
PHC encounters	Qassim	3,276,376	3,150,842	−125,534	−3.8%
PHC encounters	Riyadh	6,500,432	6,837,872	+337,440	+5.2%
PHC encounters	Tabuk	1,530,667	1,395,313	−135,354	−8.8%
OPD visits	Al Baha	616,210	495,326	−120,884	−19.6%
OPD visits	Al Jouf	449,842	517,623	+67,781	+15.1%
OPD visits	Asir	1,245,523	1,176,546	−68,977	−5.5%
OPD visits	Eastern	2,306,613	3,140,427	+833,814	+36.1%
OPD visits	Hail	295,368	471,239	+175,871	+59.5%
OPD visits	Jazan	739,865	960,201	+220,336	+29.8%
OPD visits	Madinah	873,621	1,209,078	+335,457	+38.4%
OPD visits	Makkah	2,707,884	3,414,828	+706,944	+26.1%
OPD visits	Najran	292,824	623,018	+330,194	+112.8%
OPD visits	Northern Borders	262,792	382,065	+119,273	+45.4%
OPD visits	Qassim	1,115,765	1,123,505	+7740	+0.7%
OPD visits	Riyadh	3,118,390	2,893,159	−225,231	−7.2%
OPD visits	Tabuk	504,402	389,210	−115,192	−22.8%
Inpatient admissions	Al Baha	33,241	51,708	+18,467	+55.6%
Inpatient admissions	Al Jouf	72,304	62,896	−9408	−13.0%
Inpatient admissions	Asir	114,303	93,097	−21,206	−18.6%
Inpatient admissions	Eastern	139,360	434,299	+294,939	+211.6%
Inpatient admissions	Hail	35,487	62,921	+27,434	+77.3%
Inpatient admissions	Jazan	59,140	154,405	+95,265	+161.1%
Inpatient admissions	Madinah	85,016	162,322	+77,306	+90.9%
Inpatient admissions	Makkah	227,705	362,838	+135,133	+59.3%
Inpatient admissions	Najran	46,125	73,229	+27,104	+58.8%
Inpatient admissions	Northern Borders	27,900	47,713	+19,813	+71.0%
Inpatient admissions	Qassim	101,205	102,819	+1614	+1.6%
Inpatient admissions	Riyadh	180,376	200,502	+20,126	+11.2%
Inpatient admissions	Tabuk	47,810	67,974	+20,164	+42.2%

Note: Counts represent recorded service volumes, not unique patients.

**Table 4:** National period-specific change in absolute MoH counts.

Indicator	2016–2019	2019–2020	2020–2024	2019–2024
PHC centers	−2.8%	−0.2%	−3.8%	−3.9%
MoH hospitals	+4.4%	+0.3%	+1.0%	+1.4%
MoH beds	+6.8%	+1.2%	+3.8%	+5.0%
Physicians	+29.0%	+2.0%	+35.3%	+38.0%
Nurses	+7.7%	+0.6%	+11.2%	+11.8%
PHC encounters	+9.1%	−33.4%	+14.0%	−24.1%
OPD visits	−13.7%	−37.9%	+115.7%	+33.9%
Inpatient admissions	+21.7%	−17.6%	+59.9%	+31.8%

Note: Values are descriptive percentage changes in absolute counts. The 2019–2020 column shows the marked change in recorded values during 2020 and is not a causal estimate of the pandemic.

**Table 5:** National capacity-to-utilization ratios in selected years.

Ratio	2016	2019	2020	2023	2024	2016–2024
PHC encounters per PHC center	21,427	24,035	16,030	22,500	18,996	−11.3%
OPD visits per MoH hospital	53,026	43,844	27,136	52,101	57,918	+9.2%
Inpatient admissions per MoH bed	28.0	31.9	26.0	30.0	40.0	+43.1%

Note: Ratios summarize recorded activity per facility or bed. They do not account for case mix or length of stay.

**Table 6:** Endpoint sensitivity analysis for national absolute counts.

Indicator	2016–2023 Count Change (%)	2016–2024 Count Change (%)	2023–2024 Count Change (%)
PHC centers	−8.6%	−6.6%	+2.2%
MoH hospitals	+5.8%	+5.8%	0.0%
MoH beds	+13.1%	+12.1%	−0.8%
Physicians	+52.5%	+78.1%	+16.8%
Nurses	+10.8%	+20.5%	+8.7%
PHC encounters	−4.0%	−17.2%	−13.7%
OPD visits	+4.0%	+15.6%	+11.2%
Inpatient admissions	+21.2%	+60.4%	+32.3%

Note: The 2016–2024 analysis remains primary. The comparison through 2023 assesses sensitivity to the 2024 reporting transition and endpoint changes; it does not validate equivalence of definitions across years. National and regional 2016–2023 changes and CAGRs are provided in Supplementary File S1.

## Data Availability

All source data are publicly available in the Saudi MoH Statistical Yearbooks and the KAPSARC Data Portal [2,3]. The complete harmonized analytic dataset, extraction log, correction log, 2024 crosswalk, indicator-construction rules, population denominators, derived tables, and analytical code are provided with this submission as Supplementary Files S1 and S2, permitting direct verification without a request to the corresponding author.

## Supplementary Materials

The following supporting information can be downloaded at https://www.mdpi.com/article/10.3390/healthcare14183033/s1, Supplementary File S1: Supplementary_Data_and_Analysis.xlsx (validated indicator data, extraction and correction logs, population denominators, analytic region–year file, construction rules, 2024 crosswalk, and derived descriptive tables); Supplementary File S2: Supplementary_Analysis_Code.py (code used to reproduce the population-adjusted analyses, tables, and figures).

## Abbreviations

The following abbreviations are used in this manuscript:

CAGR	Compound annual growth rate
HSTP	Health Sector Transformation Program
MoH	Ministry of Health
OPD	Outpatient department
PHC	Primary healthcare
SDG	Sustainable Development Goal

## Appendix A. 2024 Harmonization and Source Documentation

The 2024 crosswalk is based on the official MoH yearbook worksheet identified in Section 2.4. The source is an Excel workbook; therefore, worksheet/table and row references are reported instead of PDF page numbers. Each named reporting unit used in the analytic data was assigned once, making the named-unit mapping mutually exclusive. Geographic allocation was not fully exhaustive because of the unassignable PHC category. The unassignable ‘Undefined’ PHC value is disclosed separately and was not included in a regional value.

**Table A1:** Indicator-specific 2024 source tables and harmonization treatment.

Indicator	Official 2024 Source	Reporting Unit	Treatment
PHC centers	Worksheet 2-5, named units in rows 5–24 (counts F; names G)	MoH branches/offices	Summed to regions using official crosswalk
Hospitals; beds	Worksheet 2-6, named units in rows 7–26 (hospitals J; beds K; names L)	MoH branches/offices	Summed to regions using official crosswalk
Physicians; nurses	Worksheets 2-41 (hospital) and 2-44 (PHC): physician totals in rows 10, 39, 68, 97, 126; nursing/midwifery totals in rows 25, 54, 83, 112, 141. Named-unit total columns E, H, K, N in each block; dentists excluded.	MoH branches/offices	Hospital and PHC categories constructed, then summed
PHC encounters; OPD visits	Worksheet 4-6: named clusters, rows 6–25 (PHC B; OPD C); Undefined, row 26. Worksheet 4-7: regions, rows 7–19 (PHC J; OPD K); Undefined, row 20.	Clusters/regions	Named clusters assigned once; undefined PHC disclosed
Inpatient admissions	Worksheet 4-22, named clusters in rows 6–25 (admissions G; names H); national total, row 26	Health clusters	Clusters assigned once and summed to regions

Note: The full indicator-by-year extraction log is included in Supplementary File S1.

**Table A2:** Official crosswalk from 2024 branches/offices and health clusters to administrative regions.

Administrative Region	Branch/Office Units	Health-Cluster Units	Crosswalk Location
Riyadh	Riyadh	Riyadh First; Riyadh Second; Riyadh Third	Rows 3–5
Makkah	The Holy Capital; Qunfudah; Jeddah; Taif	Makkah; Jeddah First; Jeddah Second; Taif	Rows 6–10
Madinah	Madinah	Madinah	Row 11
Qassim	Qassim	Qassim	Row 12
Eastern	Eastern; Al Ahsa; Hafr Al Batin	Eastern; Al Ahsa; Hafr Al Batin	Rows 13–15
Asir	Asir; Bishah	Asir	Rows 16–17
Tabuk	Tabuk	Tabuk	Row 18
Hail	Hail	Hail	Row 19
Northern Borders	Northern Borders	Northern Borders	Row 20
Jazan	Jazan	Jazan	Row 21
Najran	Najran	Najran	Row 22
Al Baha	Al Baha	Al Baha	Row 23
Al Jouf	Al Jouf; Qurayyat	Al Jouf	Rows 24–25

Note: Source: Statistical-Yearbook-2024.xlsx, worksheet ‘Regions—English’ (English translation of the worksheet name), cells B1:F25 [3]. For branches/offices without a separately named health cluster (Qunfudah, Bishah, and Qurayyat), the official worksheet places the governorate within its administrative region; cluster-based service tables use the corresponding regional cluster category.
